# Attempted replication of SNPs in RANKL and OPG with musculoskeletal adverse events during aromatase inhibitor treatment for breast cancer

**DOI:** 10.1152/physiolgenomics.00085.2017

**Published:** 2017-12-06

**Authors:** Jacqueline M. Dempsey, Jingyue Xi, N. Lynn Henry, James M. Rae, Daniel L. Hertz

**Affiliations:** ^1^Department of Clinical Pharmacy, University of Michigan College of Pharmacy, Ann Arbor, Michigan; ^2^Department of Biostatistics, University of Michigan School of Public Health, Ann Arbor, Michigan; ^3^Huntsman Cancer Institute, University of Utah Health Care, Salt Lake City, Utah; ^4^Department of Internal Medicine, Division of Hematology/Oncology, University of Michigan Medical School, Ann Arbor, Michigan

**Keywords:** aromatase inhibitors, musculoskeletal adverse events, OPG, RANKL

## Abstract

Aromatase inhibitor (AI) therapy is highly efficacious in the treatment of estrogen receptor-positive breast cancer; however, in a subset of patients AI use is discontinued due to drug-induced musculoskeletal adverse events (MS-AE). Several studies have investigated the role of germline single nucleotide polymorphisms (SNPs) on patients’ risk of MS-AEs; however, no associations have yet to be validated for translation into clinical practice. This study attempted to replicate SNPs in RANKL (rs7984870) and OPG (rs2073618) on the risk of AI-induced MS-AEs and screen for secondary associations with MS-AE-related treatment discontinuation and serum and urine markers of bone health. Previously reported associations were not replicated with our primary hypothesis, change in MS-AE from baseline to 3 mo; however, patients homozygous for the G allele of rs7984870 in RANKL had lower risk of MS-AE-associated treatment discontinuation in analyses of secondary phenotypes without statistical correction.

## BACKGROUND/MOTIVATION FOR THE STUDY

Third-generation aromatase inhibitors (AIs) are highly efficacious in the treatment of estrogen receptor-positive (ER+) breast cancer and are a first line option in postmenopausal patients. AIs inhibit aromatase-mediated systemic production of estrogens from androgenic precursors. While many patients taking AIs achieve efficacy with tolerable side effects, their use is restricted in a subset of patients due to drug-induced musculoskeletal adverse events (MS-AEs). Several studies have attempted to discover germline single nucleotide polymorphisms (SNPs) that affect risk of MS-AE development; however, no associations have yet to be validated in independent patient cohorts. Validation of a genetic predictor of AI-related toxicity could be used to help inform selection of appropriate hormonal treatment in patients with ER+ breast cancer. This study attempted to validate previously reported associations for SNPs in RANKL and osteoprotegerin (OPG) on the risk for MS-AEs in patients receiving AIs. RANKL is responsible for the differentiation of osteoclasts leading to bone resorption. The rs7984870 SNP has been associated with RANKL expression and the minor (G) allele reported by Wang et al. (5) for an exclusively Asian cohort was protective for MS-AEs. The rs2073618 SNP has been associated with expression of OPG, a decoy receptor of RANKL. Wang et al. found the minor (G) allele to be protective for MS-AE’s, whereas Lintermans et al. (4) reported highest risk of MS-AEs in the heterozygous group (CG). These conflicting results (see supplemental material: Appendix 2, Table 3) warrant additional replication in independent cohorts to validate these associations for potential translation into clinical practice. (For figure and all tables, see this article’s supplemental material.)

## PHENOTYPE

The primary phenotype was the change in musculoskeletal (MS) symptom cluster from baseline to 3 mo of AI therapy as previously defined (3). Briefly, patients self-reported their side effects and the severity on a scale from 0 to 4. The symptom cluster score was the square root of the sum of arthralgias, myalgias, joint pain/stiffness, tendinitis, numbness/tingling, and/or carpal tunnel syndrome. The secondary phenotype of interest included the time to discontinuation of AI therapy due to MS-AEs as previously defined (1). Changes in serum and urine biomarkers of bone turnover markers were also screened for pharmacologic associations.

### 

#### Cohort details.

The current study was a secondary pharmacogenetic analysis conducted in a cohort of 500 postmenopausal women with stage I–III hormone-receptor positive breast cancer prospectively enrolled in the previously described Exemestane and Letrozole Pharmacogenetics Trial (2). Patients were randomly assigned to exemestane 25 mg/day or letrozole 2.5 mg/day orally for up to 2 yr. The majority of subjects (89%) were Caucasian, with the remaining patients primarily of African American or Asian descent. Additional demographic information is available in previous publications (2).

#### Type of study.

Candidate SNP replication.

#### Details of the SNPs studied.

rs7984870 and rs2073618.

#### Analysis model.

A linear regression model assuming an additive genetic effect was used to analyze univariate associations between each SNP and each phenotype of interest. Change in MS-AE from baseline to 3 mo was selected as the primary phenotype and analyzed against a significance threshold of *P* < 0.05. Other phenotypes (time to MS-AE-related AI discontinuation, serum/urine biomarkers of bone health) were included as secondary, hypothesis-generating analyses. Significant findings for any univariate association were adjusted for treatment arm and tested within each treatment arm to determine whether associations were AI specific or shared.

## RESULTS

The rs7984870 variant [RANKL, chromosome 3, position 43;146,482, minor (C) allele frequency = 0.45, Hardy Weinberg Equilibrium (HWE) *P* = 0.40] was not associated with change in MS-AE from baseline to 3 mo (*P* = 0.173, Table 2). In the secondary analysis, rs7984870 was significantly associated with time to MS-AE-related discontinuation of AI therapy (β = 0.309, STD = 0.139 *P* = 0.0261, Fig. 1). This association maintained significance after adjustment for treatment arm (*P* = 0.0321), and the direction of association was consistent in the exemestane and letrozole arms, though neither analysis maintained independent significance (both *P* > 0.05). Although the minor allele frequency of rs7984870 varies across ancestral populations (Table 1), significance was maintained in an analysis limited to Caucasians (*P* = 0.0015, Table 4). The rs2073618 variant (OPG, chromosome 8 position 119,964,052, minor (G) allele frequency = 0.49, HWE *P* = 0.50) was not significantly associated with change in MS symptom cluster (*P* = 0.134) or any secondary phenotypes (all *P* > 0.05, Table 2).

## INTERPRETATION

In our uncorrected secondary analysis, the G allele of rs7984870 (RANKL) was nominally associated with protection from MS-AE-related AI discontinuation. These findings are consistent with the protective effect of the G allele on occurrence of an MS-AE symptom cluster (joint pain, muscle pain, bone pain, arthritis, diminished joint function, or other musculoskeletal problems) previously reported by Wang et al. (Table 3) (5). Study limitations include the lack of significance of our preselected primary phenotype, the lack of statistical correction employed for the detected association (Table 2), between-study differences in MS-AE collection and definition, potential confounding from analyzing a multiancestry cohort, and the lack of RANKL and OPG expression data in our cohort.

## GRANTS

This research was supported by Pharmacogenetics Research Network Grant U-01 GM-61373 (D. A. Flockhart) and Clinical Pharmacology Training Grant 5T32-GM-08425 (D. A. Flockhart) from the National Institute of General Medical Sciences (NIGMS), National Institutes of Health (NIH), from Grants M01-RR-000042 (University of Michigan), M01-RR-00750 (Indiana University), and M01-RR00052 (Johns Hopkins University) from the National Center for Research Resources, a component of the NIH, the Breast Cancer Research Foundation (N003173 to J.M.R. and D. F. Hayes), the National Cancer Institute (5T32CA-083654-12, PI Jeremy Taylor), the NIGMS (GM-099143 to J.M.R.), and the NIH through the University of Michigan’s Cancer Center Support Grant (P30 CA-046592) by the use of the following Cancer Center Core: University of Michigan DNA Sequencing Core. In addition, these studies were supported by grants from Pfizer (D. F. Hayes), Novartis Pharma AG (D. F. Hayes), the Fashion Footwear Association of New York/QVC Presents Shoes on Sale (D. F. Hayes). Drugs were supplied by Novartis and Pfizer.

## DISCLOSURES

No conflicts of interest (financial or otherwise) are declared by the authors.

## AUTHOR CONTRIBUTIONS

J.M.D., N.L.H., J.M.R., and D.L.H. conceived and designed research; J.M.D., N.L.H., and D.L.H. interpreted results of experiments; J.M.D. drafted manuscript; J.M.D., N.L.H., J.M.R., and D.L.H. edited and revised manuscript; J.M.D., J.X., N.L.H., J.M.R., and D.L.H. approved final version of manuscript; J.X. analyzed data; J.X. prepared figures.

## Supplemental Data

Figure 1 Figure 1 - .pdf (110 KB)

Table 1 Table 1 - .xlsx (10.5 KB)

Table 2 Table 2 - .xlsx (12.6 KB)

Table 3 Table 3 - .docx (14.8 KB)

Table 4 Table 4 - .xlsx (12.6 KB)
