# Supplementary figures and images for: Attempted replication of SNPs in RANKL and OPG with musculoskeletal adverse events during aromatase inhibitor treatment for breast cancer
Source: Physiol Genomics. 2017 Dec 6;50(2):98–9. doi: 10.1152/physiolgenomics.00085.2017 (PMC5867615; doi:10.1152/physiolgenomics.00085.2017)

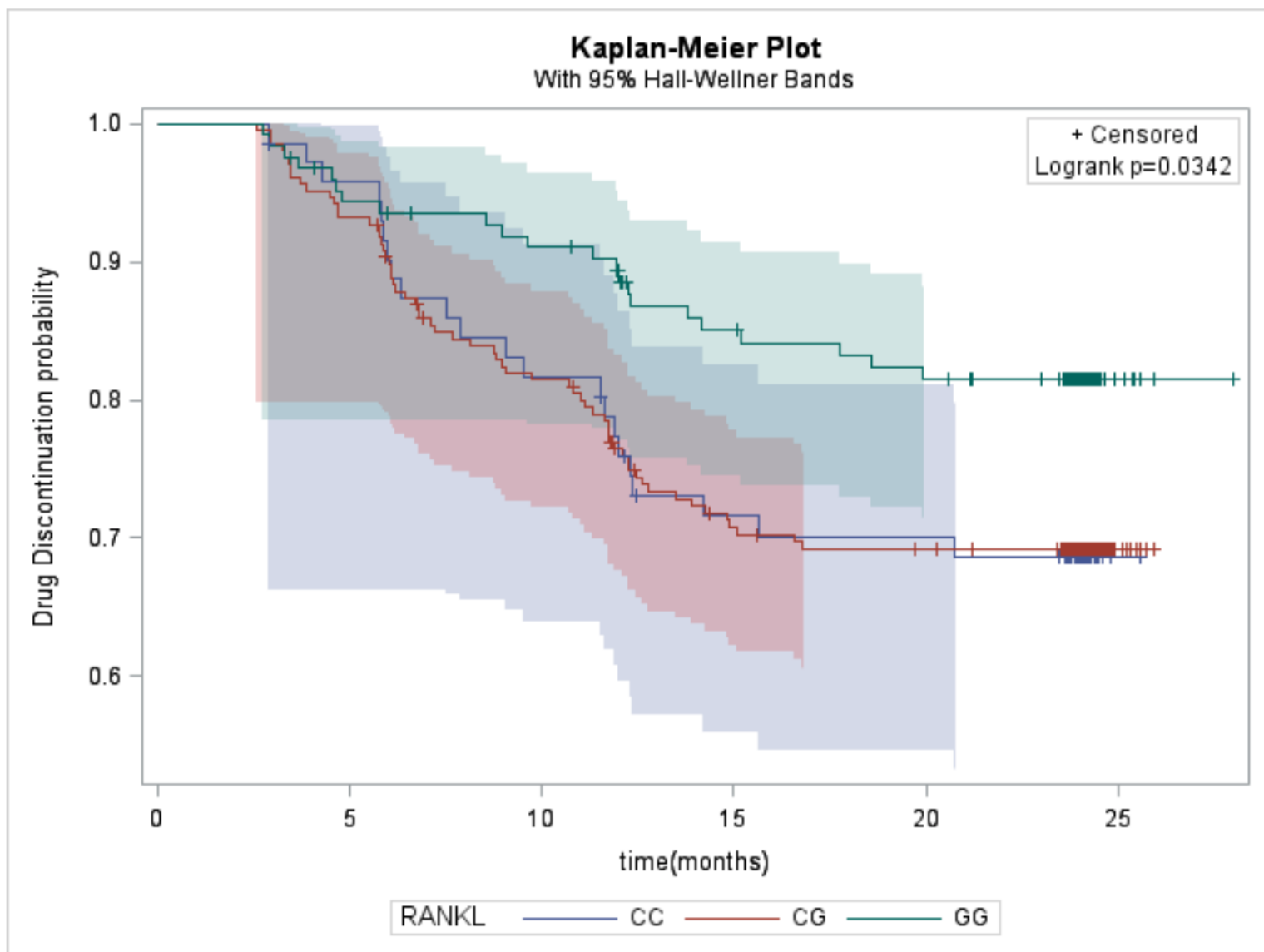

Figure 1. Time to Discontinuation probability by RANKL (CC vs CG vs GG)

Supplement: Figure 1 — pdf (110 KB) [file figure1.pdf]
