# Supplementary material for: Attempted replication of SNPs in RANKL and OPG with musculoskeletal adverse events during aromatase inhibitor treatment for breast cancer
Source: Physiol Genomics. 2017 Dec 6;50(2):98–9. doi: 10.1152/physiolgenomics.00085.2017 (PMC5867615; doi:10.1152/physiolgenomics.00085.2017)
Supplement: Table 3 — docx (14.8 KB) [file table3.docx]

| Study | Reference SNP ID | Genotype | n | Cases^a^ | Odds Ratio (95% CI)^b^ | P value^c^ |
| --- | --- | --- | --- | --- | --- | --- |
| Wang et al. | rs7984870 (RANKL) | CC | 119 | 76 (64%) | 3.26 (1.84 - 5.76) | 2.19E-04 |
|  |  | CG | 210 | 100 (48%) | 1.68 (1.01 - 2.79) | 0.76 |
|  |  | GG | 91 | 32 (35%) | Reference | 2.00E-03 |
|  | rs2073618 (OPG) | GG | 142 | 59 (42%) | Reference | 2.30E-02 |
|  |  | GC | 204 | 99 (49%) | 1.33 (0.86 - 2.04) | 0.697 |
|  |  | CC | 74 | 50 (68%) | 2.93 (1.62 - 5.29) | 7.95E-04 |
| Lintermans et al. | rs2073618 (OPG) | CC | 48 | 30 (63%) | Reference | 0.046 |
|  |  | CG/GG | 106 | 84 (86%) | 2.29 (1.08-4.85) |  |

Table 3

^a^: Cases were defined in Wang et al as the presence of at least grade 3 toxicity according to NCI CTCAE V3. for joint pain, muscle pain, bone pain, arthritis, diminished joint function, or other musculoskeletal problems. Cases were defined in Lintermans et al as an increase in arthralgia or myalgia from baseline.

^b^: Odds ratios and confidence intervals reported as presented in Wang et al. Odds ratios and confidence intervals were not reported by Lintermans et al., so they were estimated using an online calculator (https://www.medcalc.org/calc/odds_ratio.php)

^c^: P-values from Wang et al. are the comparison of the genotype frequencies in cases vs. controls. P-value from Lintermans et al. is the proportion of carriers of the minor (G) allele who were cases, compared with the proportion of wild-type (CC) patients who were cases (i.e. dominant genetic model).
